# Urinary catheters: state of the art and future perspectives – a narrative review

**DOI:** 10.1016/j.mtbio.2025.102225

**Published:** 2025-08-20

**Authors:** Kristijan Skok, Uroš Bele, Špela Pintar, Zdenka Peršin, Katja Kuzmič, Matej Bračič, Lidija Fras Zemljič, Uroš Maver

**Affiliations:** a Diagnostic and Research Institute of Pathology, Medical University of Graz, Neue Stiftingtalstraße 6, 8010, Graz, Austria; b University of Maribor, Faculty of Medicine, Institute of Biomedical Sciences, Taborska Ulica 8, SI-2000, Maribor, Slovenia; c Department of Urology, Medical University of Graz, Neue Stiftingtalstraße 6, 8010, Graz, Austria; d Faculty of Medicine, University of Ljubljana, Ljubljana, Slovenia; e Department of Gastroenterology, University Medical Centre Ljubljana, Ljubljana, Slovenia; f University of Maribor, Faculty of Mechanical Engineering, Smetanova Ulica 17, SI-2000, Maribor, Slovenia; g University of Maribor, Faculty of Medicine, Department of Pharmacology, Taborska Ulica 8, SI-2000, Maribor, Slovenia

**Keywords:** Urinary catheters, Urinary tract infection, Antibiotic resistance, Nanotechnology, Biomedical engineering

## Abstract

Catheter associated urinary tract infection (CAUTI) is the most frequent healthcare associated infection, arising from microbial adhesion to catheter surfaces, biofilm development, and the growing problem of antimicrobial resistance. Many publications have addressed CAUTI epidemiology, biofilm biology, or biomaterials for catheters in isolation, yet there is little literature that connects these areas into a coherent translational perspective. This review seeks to fill that gap by combining an overview of biofilm pathophysiology with recent advances in material based innovations for catheter design, including nanostructured and responsive coatings, sensor enabled systems, additive manufacturing, and three dimensional printing. Established approaches such as hydrophilic or antimicrobial impregnated catheters are considered alongside bio inspired surface textures, zwitterionic polymers, and multifunctional hydrogels. Each strategy is evaluated in terms of maturity, clinical applicability, and barriers to translation, with a focus on shifting from antibiotic dependent treatment toward prevention of biofilm formation. By bringing together knowledge from microbiology, engineering, and clinical urology, the review outlines pathways for developing the next generation of catheters that improve outcomes and reduce infection rates.

## Graphical abstract

**Figure:**
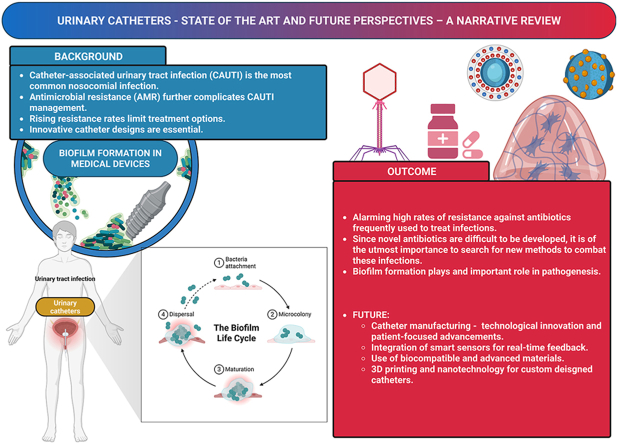

## 1 Introduction

Around the world, catheter-associated urinary tract infection (CAUTI) is the leading type of hospital acquired infection [1]. It accounts for 20–40 % of nosocomial infections in the US and Europe [2] with a mortality rate of ca. 10 % [3]. It's estimated that between 15 % and 25 % of individuals admitted to hospitals undergo urethral catheterization during their time there [4–6]. This percentage is even higher in an intensive care setting [3,4]. With more than 30 million bladder catheters inserted in the US every year, CAUTI affects hundreds of thousands of patients [5]. Extended use of a urinary catheter is the primary factor that increases the likelihood of developing a CAUTI with the incidence of bacteriuria of 3–8 % per day [3]. Urinary tract infections (UTIs) are classified as either uncomplicated or complicated, depending on whether there are underlying conditions that disrupt the normal function of the urinary tract [6].

Urinary catheterization can be divided based on the location (suprapubic or transurethral) or duration (intermittent, indwelling). There's an apparent increase in the placement of indwelling urinary catheters, and most individuals in hospitals are catheterized for a period ranging from two to four days [7]. Many urinary catheters are placed unnecessarily [8], can remain in use unbeknownst to the doctor [8], aren't taken out as soon as they're no longer required, and cause both discomfort and mobility restrictions for patients [9]. In standard hospital settings, it's reported that 30 % of initial urinary catheter placements lack proper justification [2]. CAUTI prevention has become a key focus for many hospitals, as studies suggest 65 %–70 % of these infections can be avoided [9]. US data indicates that 95.483 to 387.550 CAUTIs could be prevented each year, potentially saving 2.225 to 9.031 lives annually [7]. An individual CAUTI event in the US is projected to cost no less than $600, and a bloodstream infection linked to the urinary tract costs at least $2.800 ^9^. According to data from the European Center for Disease Prevention and Control, an estimated 4.1 million individuals contract healthcare-associated infections each year while receiving treatment in hospitals across Europe [10]. Among these infections, urinary tract infections (UTIs) encompasses around 30 %. The estimated annual costs of preventable CAUTIs range, depending on the source, from $115 million to $1.8 billion [11]. Hollenbeak et al. wanted to specify the attributable cost of CAUTIs. Their findings indicated a wide range in CAUTI costs, which they attributed to differences in patient demographics, severity of illness, and the chosen financial viewpoint. They concluded that attributable costs likely exceed $1.000 [12]. CAUTIs are often clinically unapparent (e.g., catheter-associated asymptomatic bacteriuria; CAASB) and with a benign course [7,13]. However, in some patients, catheter-associated bacteriuria/funguria (CABF) can lead to serious consequences (e.g., urosepsis, organ failure, death), and may therefore be associated with excess mortality. All in all, it comes as no surprise that CAUTI has significant clinical and economic consequences [2].

**Fig. 1:**
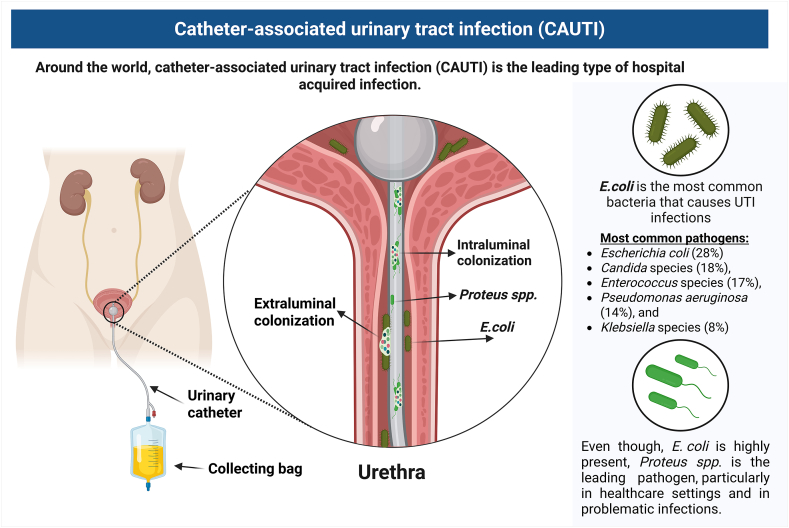
Depiction of a urinary catheter and common CAUTI associated pathogens. Created with *Biorender. com.*

Dealing with these infections is particularly difficult because pathogens can readily cling to the catheter material, forming biofilms that grow on both inanimate and biological surfaces (Fig. 1) [10,14,15]. Microbial biofilms are also a major factor, involved in about 80 % of human microbial infections [10]. These biofilms provide a reservoir for pathogens, act as an adhesive foundation, and defend the embedded cells against detachment by flow shear [15]. In using catheters, they can block its lumen, which causes obstruction and can ultimately lead to kidney infection and septicemia [14,16,17]. To tackle this problem, a multitude of methods have been described. These include already established systems (e.g., the use of secured, closed silicone urinary catheter drainage systems, reminder systems [18], etc.) as well as novel strategies focused on the biocompatibility of catheter materials as well as coating designed to combat infections [7,19]. The most common pathogens causing CAUTIs originate primarily from the patient's own microbiota, although environmental sources within the hospital setting may also contribute. In females, enteric bacteria often colonize the periurethral area due to the short anogenital distance, facilitating ascent into the urinary tract (Fig. 1) [20]. According to the European Centre for Disease Prevention and Control (ECDC), the most frequently isolated CAUTI pathogens are *Escherichia coli* (28 %), Candida spp. (18 %), Enterococcus spp. (17 %), *Pseudomonas aeruginosa* (14 %), and Klebsiella spp. (8 %) [21]. More on those in chapter 2.2. Biofilm formation on urinary catheters follows five sequential stages. These changes and the process in detail will be discussed in chapter 2.4.3 and 2.4.4. The biofilm acts as a protective niche, promoting persistence, antimicrobial tolerance, and recurrent infection.

While several reviews discuss CAUTI epidemiology, biofilm biology, or biomaterial developments separately, few synthesize these domains into an integrated framework. This review uniquely bridges clinical urology with advances in materials science, including nanostructured surfaces, responsive hydrogels, smart coatings, and additive manufacturing, placing these innovations in the context of biofilm pathophysiology and the growing threat of AMR. By combining insights from microbiology, engineering, and clinical practice, we aim to outline translational pathways toward next‐generation catheter technologies.

## 2 Urinary infections

### 2.1 Basic nomenclature

**Fig. 2:**
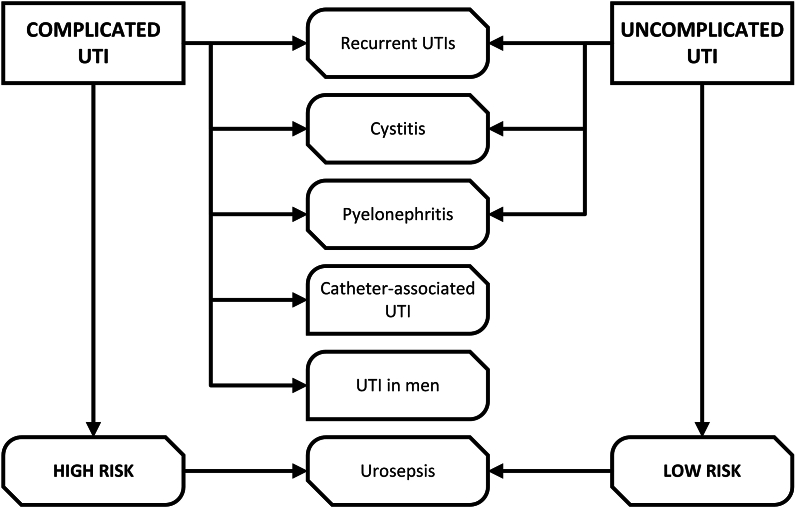
The concept of uncomplicated and complicated UTI. Summarised after the current guidelines from Bonkat et al. [3].

The concept of uncomplicated versus complicated UTI is commonly found in guidelines. (Fig. 2). Due to in literature often misused nomenclature, we start with the most common definitions.

- **Infections present on admission (POA)** are not categorized as healthcare-associated infections (HAIs) [22].
- **Healthcare-associated infections (HAIs)**: An infection is considered to have occurred if it manifests on or after the third calendar day of a patient's admission, with the admission day counted as day one [22].
- **(Symptomatic) urinary tract infections ((S)UTI)**: At least one of the following symptoms is present in the patient: a fever above 38.0 °C, suprapubic tenderness, or pain/tenderness in the costovertebral angle (with no other discernible cause), urinary urgency, increased urinary frequency, dysuria (not applicable if a catheter is in place); in the patient's urine culture, a bacterium (≥10^5^ colony-forming units per millilitre (CFU/ml)) is present, and no more than two species of organisms are identified in total. Urinary tract infections are generally classified into distinct types: cystitis, which affects the bladder and lower urinary tract; pyelonephritis, involving the kidneys and upper urinary tract; and prostatitis, which targets the prostate gland [14,22].
- **Asymptomatic bacteremic UTI (ABUTI) –** The presence of over 100,000 CFU/ml in voided urine in individuals showing none of the described UTI symptoms defines this condition [22].
- **Date of event (DOE)**: The Infection Window Period (7 days) begins on the date when the first element fulfilling the UTI infection criterion initially occurred [22].
- **Indwelling catheter**: A catheter inserted through the urethra into the bladder remains in place and is connected to a drainage system, such as a leg bag or a catheter valve. Commonly referred to as Foley catheters, these are distinct from other types like condom catheters or single-use intermittent (in-and-out) catheters. Devices such as nephrostomy tubes, ureteral stents (used in procedures like ileal conduits or ureterocutaneostomies), and suprapubic catheters are generally excluded from consideration—unless a Foley catheter is also in use at the same time. Importantly, urethral catheters used for either continuous or intermittent bladder irrigation are included within the scope of CAUTI monitoring [22].
- **CAUTI**: A urinary tract infection is considered catheter-associated if an indwelling urinary catheter was used for over two calendar days by the date the infection was noted (counting the placement day as day one) [23], and the catheter was still in place on that day or the day before. If the indwelling urinary catheter had been in place for more than two days and was subsequently removed, the UTI must have occurred on the day it was taken out or the very next day to qualify as catheter-associated [22].
  o **Catheter-associated bacteriuria** (**CAB)**: A catheter urine sample showing bacterial growth of at least 10^5^ CFU/mL in adults—or 10^4^ CFU/mL in pediatric patients—of one or more bacterial species is considered clinically significant [7, 23]. In patients with long-term indwelling bladder catheters, the presence of bacteriuria is nearly inevitable and is commonly observed in individuals who have been catheterized for more than one week [22, 24], with the incidence of bacteriuria of 3–8 % per day [3].” was replaced as “The text defines this as having at least 10ˆ5 CFU/ml (or 10ˆ4 in pediatric cases) of one or more bacterial species in a single catheter urine sample. This condition is nearly universal in patients with long-term indwelling bladder catheters and is detected in most patients catheterized for over a week, with a bacteriuria incidence of 3–8 % daily [3].
- **Complicated UTI (cUTI) –** A urinary tract infection is classified as complicated when it occurs in the presence of factors that make eradication more challenging than in uncomplicated cases. These may include host-related conditions such as diabetes, immunosuppression, or pregnancy, as well as structural or functional abnormalities of the urinary tract—like obstruction or impaired bladder emptying. Additional risk factors include male sex, the use of an indwelling catheter, underlying kidney disease, or any condition that weakens immune defenses [3].
- **Urosepsis -** is defined as a severe and potentially life-threatening condition that occurs when an infection originating in the urinary tract or male reproductive organs causes an excessive and uncontrolled immune response, resulting in organ dysfunction [3].

In patients with indwelling transurethral or suprapubic catheters, or those who use intermittent bladder catheterization, a CAUTI is identified by the presence of significant bacterial growth in the urine along with symptoms of a UTI, provided no other source of infection is apparent. Importantly, the bacterial count considered clinically relevant in catheterized individuals is lower. It is set at 10^3^ CFU/mL or more of one or more bacterial species than in those without catheters [7].

### 2.2 Aetiology

The most common CAUTI pathogens are derived from the patients’ own microbiota, sometimes also from the hospital setting. Enteral bacteria often colonize the area around the external urethral orifice, especially in females due to the female anatomy (i.e. short anogenital distance) [9]. The European The European Center for Disease Prevention and Control (ECDC) identified the most commonly isolated pathogens as *Escherichia coli* (28 %), *Candida* species (18 %), *Enterococcus* species (17 %), *Pseudomonas aeruginosa* (14 %), and *Klebsiella* species (8 %) [24]. The most current data regarding antimicrobial resistance can be viewed in the annual Antimicrobial Resistance in the EU/EEA report from 2023 [21]. Moreover, in the intensive care unit setting, 12,7 % of all patients who stayed more than one day suffered at least one intensive care unit (ICU)-acquired HAU. Of the patients studied, 3 % developed a urinary tract infection. Notably, 95 % of these UTI cases occurred in individuals with urinary catheters. The most commonly identified pathogens in these catheter-associated infections were *Escherichia coli* (27.9 %), *Enterococcus* species (23.0 %), *Pseudomonas aeruginosa* (14.3 %), and *Klebsiella* species (12.6 %) [25].

Even though, *E. coli* is highly present, *Proteus* spp. is the leading pathogen, particularly in healthcare settings and in problematic infections [6]. *Proteus mirabilis* CAUTIs are especially concerning as they often involve biomineralization, potentially obstructing the catheter's lumen [7,10,15,19]. It has, due to its resilient nature and factors for biofilm formation (e.g., mannose-resistant fimbriae, capsules and urease), earned the name “the master of both adhesion and motility” [26]. To further illustrate its importance, the name (*Proteus)* stems from a shapeshifting figure in Homer's Odyssey known for evading capture [27]. It is worth mentioning that the microbiome of urinary catheters differs depending on the duration of catheterization. As a rule of thumb, short period usage (up to 7 days) -frequently colonized by a single species [10]; long-term catheterization (>30 days) - polymicrobial infection [7,28,29]. It was observed that Gram-positive cocci infections (e.g., staphylococcal and enterococcal) were mostly extraluminal, while Gram-negative infections showed an approximately even distribution across both infection pathways [7]. Hola et al. also found that during prolonged catheterization, catheters are often colonized by three or more different microorganisms, with Gram-negative bacteria being the predominant type [30]. Only 12.5 % of the infections were monomicrobial [30] One major difficulty in diagnosing and identifying the true causative pathogen is that urine cultures taken from catheters may not accurately reflect the variety of microorganisms residing within the catheter's biofilm. Microbes embedded in biofilms differ in behavior and appearance from the free-floating (planktonic) bacteria found in urine, and they frequently fail to grow effectively on conventional agar culture media [14,22,31]. Additionally, it has been stated that the administration of antibiotics as a preventive measure for infection can cause a false-negative result [10]. To better recover and quantify microorganisms attached to urinary catheters, sonication-based methods have been suggested [30]. As previously mentioned, the microbial communities may differ based on their location (inner or outer surfaces of a catheter). Incorporating a sonication step prior to culturing helps to more thoroughly detect and measure the microorganisms present on both surfaces of the catheter [33]. The microbial communities collected can then be analyzed using different techniques (e.g., traditional cultivation methods, microscopy, etc.) [34].

### 2.3 Risk factors

Evidence suggests that the most important risk factor for CAUTI still presents prolonged catheterization [32]. Other mentioned risk factors are female sex (especially heavy bacterial colonization of the perineum), advanced age, prolonged catheterization, impaired immunity, diabetes, renal dysfunction, higher severity of illness, serum creatinine level greater than 2 mg/dL at the time of catheterization and lack of antimicrobial exposure [32,33]. Failure to maintain aseptic technique during catheter insertion and care has been linked to a higher likelihood of bacteriuria [9,34]. Chenoweth et al. [9] found that indwelling urinary catheters are involved in up to 95 % of urinary tract infections among ICU patients. The risk of developing bacteriuria increases daily during catheterization, with an average rise of 3 %–10 % per day [9,29,35], . Additionally, about 20 % of patients become colonized with bacteria at the time of catheter placement, through either internal or external pathways [2]. To put it simply, roughly 26 % of patients with catheters in place for 2–10 days will develop bacteriuria, and nearly all patients with catheters lasting 30 days or more will experience bacteriuria [11].

### 2.4 Pathogenesis

#### 2.4.1 Anatomical and physiological disruption

Under normal conditions, the bladder distends as urine flows in until the intravesical pressure hits a specific threshold [36]. This process triggers both voluntary and involuntary relaxation of the urethral sphincters, allowing the bladder to empty almost completely and flush the urethra, which helps prevent infections from ascending [7,36,37]. In contrast, urinary catheterization (UC) disrupts the bladder's natural defense mechanisms and normal function [17]. The catheter exerts pressure on the urethra, reducing blood flow to the mucosa, damaging the urothelial lining, introducing microorganisms directly, and interfering with mucin production by the periurethral glands [7,38]. Additionally, instead of the normal intermittent flow during urination, an indwelling catheter creates a continuous but low-flow stream that does not efficiently cleanse the urethra [7,17]. Due to fluid pooling around the catheter balloon and the potential for airlocks caused by patient movement or manipulation, complete bladder emptying is often hindered [39]. These factors contribute to an environment that supports microbial growth [7]. Microbes usually travel to the bladder via the space between the urethral mucosa and the outer layer of the urinary catheter (extraluminal path) [40]. In case of inappropriate handling the microbes can also travel via the internal lumen of the catheter (intraluminal path) [40,41]. This contamination can occur through external sources such as the perineum or the hands of healthcare personnel, from the backward flow of contaminated urine from the collection bag into the bladder, or due to breaks in the closed drainage system. The balance between infections originating inside the catheter lumen (intraluminal) versus those occurring on its outer surface (extraluminal) largely depends on the level of care and attention given during handling of medical equipment, which can vary widely. One study reported that approximately 66 % of catheter-associated infections were extraluminal, while 34 % were intraluminal [7,41].

#### 2.4.2 Microbiological disruption and biofilm formation

**Fig. 3:**
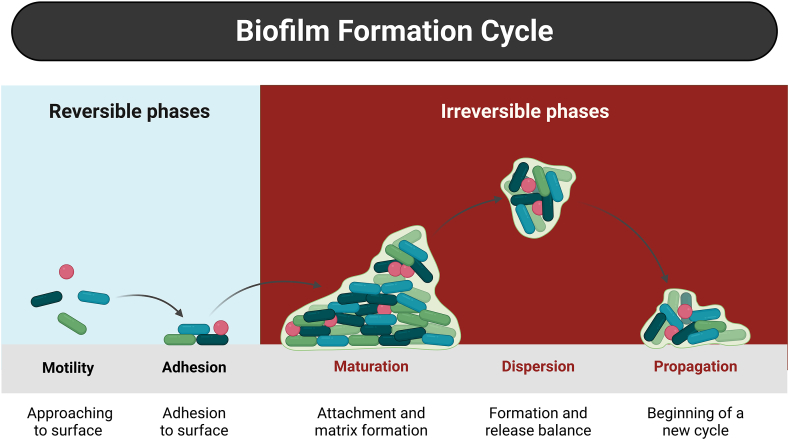
Process of biofilm formation. Legend: a) reversible attachment (adherence) of planktonic bacteria to surfaces; b) irreversible attachment to surfaces; c) cell to cell adhesion and formation of complex biomolecules; d) mature 3D biofilm structure; d) detachment and dispersal. Created with *Biorender. com*.

The formation of uropathogenic biofilms begins with the insertion of the urinary catheter. Once in place, the catheter's surface becomes coated with a conditioning film made up of various components found in urine, including proteins, organic molecules, and ions such as magnesium and calcium. This film creates a favorable environment for microbial attachment and biofilm development [7,10,42]. The alteration of the catheter surface by this process makes it an ideal site for fimbrial attachment by bacteria and uropathogenic microorganisms [7,10,42,43]. Biofilm development (Fig. 3) generally progresses through five distinct stages. It begins with the attachment of free-floating (planktonic) bacteria to a surface—initially a reversible interaction that later becomes stable and irreversible. Microorganisms in their planktonic state adhere to surfaces, utilizing either physical forces (e.g., van der Waals forces) or specific adhesion molecules like adhesins [10,44,45]. This is followed by the accumulation of a conditioning layer and the secretion of extracellular polymeric substances (EPS), which collectively form the biofilm's protective matrix. As the biofilm matures, it becomes structurally complex and resilient. The final stage involves the detachment or dispersal of bacterial cells, enabling colonization of new surfaces [44]. The transition from planktonic to biofilm-associated life forms depends heavily on the production of adhesins and matrix components that regulate each phase of biofilm formation [46,47]. Microfluidics-based flow cell systems are commonly employed to observe and study this process, offering valuable insights in both medical and environmental microbiology [46].

**Fig. 4:**
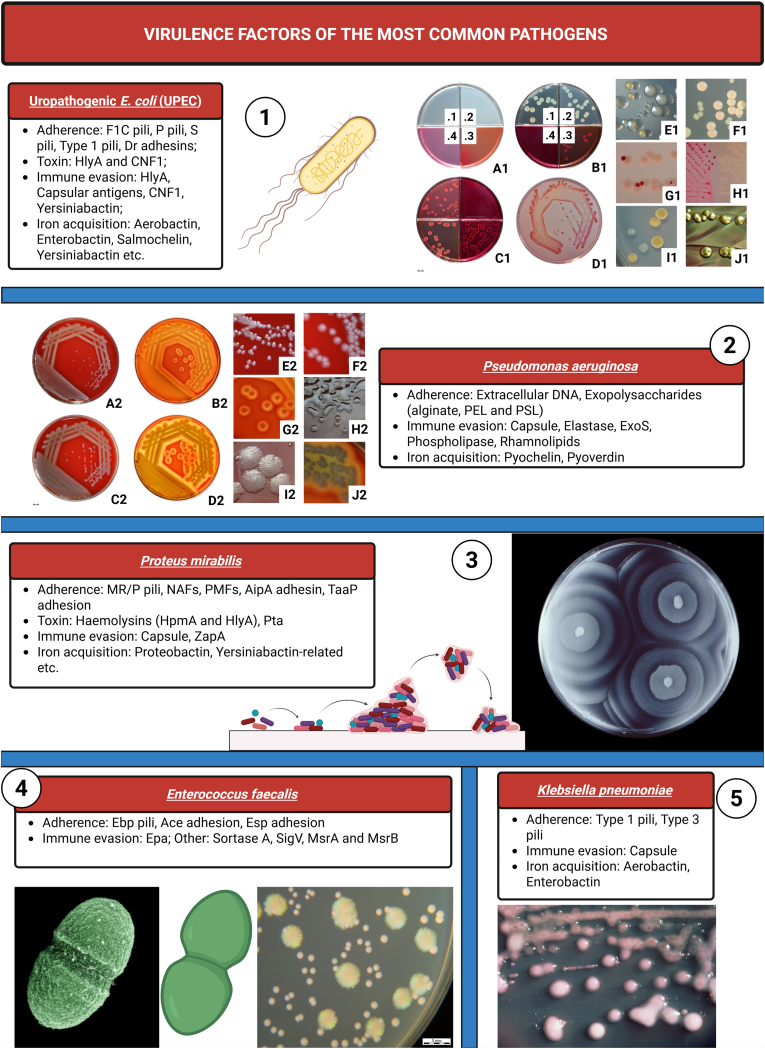
Some common pathogens and their virulence factors Legend: *Escherichia coli* grows on many commonly used cultivation media. Examples of these media are: Panel 1 – A1.1 Brain heart infusion agar (BHIA), A1.2 Trypticase soy agar (TSA), A1.3 MacConkey agar (MCA), A1.4 Endo agar. The panel B1 shows growth of a common *E. coli* strain on these media. Panel 2 - *Pseudomonas aeruginosa* growing on sheep blood agar at 37 °C after 24 h (A2, C2, E2, F2) and 48 h (B2, D2, G2, H2, I2, J2). *P. aeruginosa* strains are frequently haemolytic (B2, D2, G2, J2; reflected + transmitted light). This species gives rise to a variety of colony types. Panel 3 – TSA growth medium, that had been inoculated with *Proteus mirabilis*. Panel 4 – (left side) *Enterococcus faecalis* and (right side) *Pseudomonas aeruginosa* and *Enterococcus faecalis* on TSA. Panel 5 - MacConkey agar culture plate with *Klebsiella pneumoniae*. Created in https://BioRender.com. Reused from: https://commons.wikimedia.org; licensed under the Creative Commons Attribution-Share Alike 4.0 International license or licensed as public domain; Authors: Dr. John J. Farmer – CDC and HansN.

Other well-known virulence factors (Fig. 4) are flagella and pili facilitate the initial binding of microorganisms to uroepithelial cells and the catheter's surface [48]. Uropathogens can multiply and overcome the host's immune defences, allowing them to advance to the kidneys. There, they re-adhere using adhesins or pili to colonize the renal epithelium and then produce toxins that harm tissues [48].

#### 2.4.3 Biofilm properties

Biofilm is much more than just a layer of “slime” encasing microorganisms. It represents a structured, microbiologically derived community in which cells are attached to each other and to surfaces—whether living tissue or inert material [10,14]. Biofilms are composed of bacterial cells arranged into structured, non-randomly distributed microcolonies, which typically occupy about 15–20 % of the total biofilm volume. These microcolonies are embedded within a self-produced matrix of EPS, which provides structural support and protection to the bacterial community [35]. Accounting for about 80–90 % of the biomass [49], the matrix shows distinct characteristics in terms of both its growth rate and gene transcription. It also offers protection against detachment by flow shear [14].

The physical characteristics of a biofilm, such as its structure, stability, and adherence, are primarily governed by the composition of the EPS. In contrast, the physiological behavior of the biofilm, including nutrient processing, resistance mechanisms, and metabolic activity, is regulated by the bacterial cells within the matrix [15]. Within the biofilm, bacteria are embedded in a viscous/sticky mesh of EPS, primarily composed of polysaccharides. These tangled fibers serve to anchor the cells both to each other and to the surface, forming a cohesive and protective community. This structural arrangement not only affects the mechanical stability of the biofilm but also significantly influences microbial behavior, including communication, nutrient access, and resistance to external stress. The specific types of polysaccharides present vary depending on the microbial species and even the strain. For instance, *Pseudomonas aeruginosa* produces alginate, staphylococci generate polysaccharide intercellular adhesin (PIA), while *Streptococcus* and *Lactobacillus* species synthesize glucans and fructans [15]. Moreover, polysaccharides play a critical role in numerous biofilm functions, including initial surface attachment, detachment dynamics, structural integrity, intercellular communication and interaction, horizontal gene transfer, stress tolerance, and resistance to antibiotics.

**Fig. 5:**
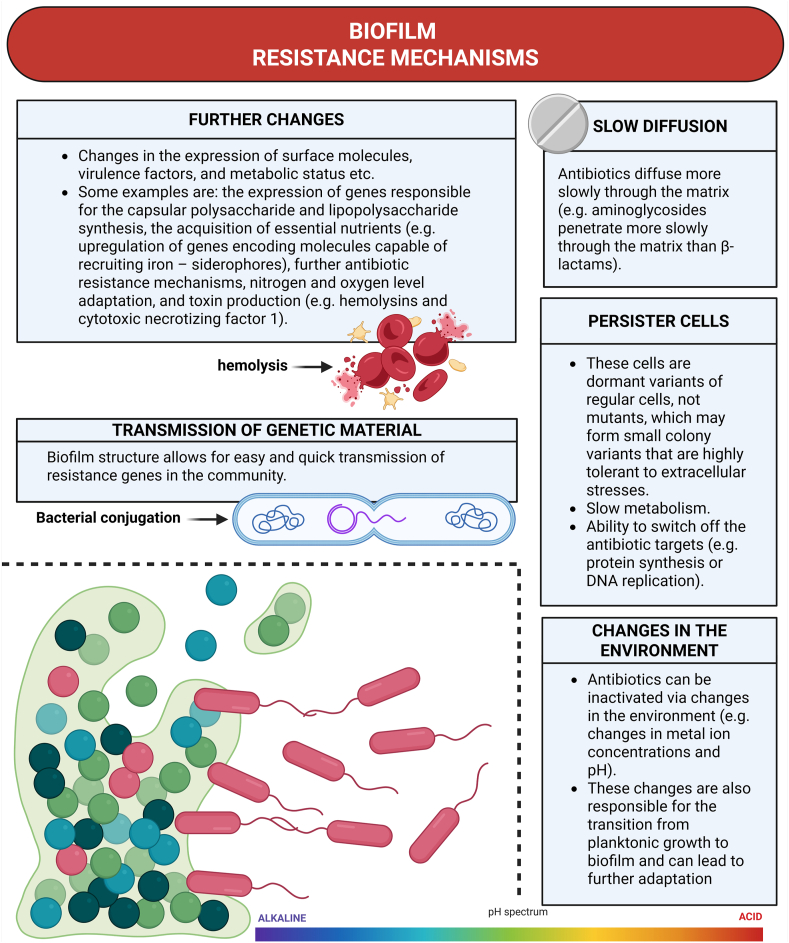
Biofilm resistance mechanisms overview. Legend: Overview of key biofilm resistance mechanisms in CAUTI, including reduced antibiotic penetration, genetic exchange, metabolic and phenotypic adaptation, persister cell formation, and environmental modifications (e.g., pH shifts). The pH spectrum highlights the frequent neutral to alkaline conditions in urease‐producing infections. Adapted from Khatoon et al. [50]. Created in https://BioRender.com.

Research indicates that biofilm bacterial cells generally require significantly higher minimum inhibitory concentrations (MICs) and minimum bactericidal concentrations (MBCs) – approximately 10–1000 times greater – than their planktonic counterparts. This means that traditional antibiotic therapy cannot reach the necessary effective MBC *in vivo* for biofilm eradication. The reason being high concentrations of medication traverse the safety window and enter the area of toxic side effects, which frequently manifest themselves in hepatic and renal toxicity. This survivability and resilience of biofilm bacteria can be attributed to multiple different mechanisms presented in Fig. 5 [14].

Firstly, antibiotics penetrate the biofilm matrix at a reduced rate—for instance, aminoglycosides diffuse more slowly than β-lactams [14]. Secondly, the architecture of the biofilm facilitates rapid and efficient transfer of resistance genes among bacterial cells. A third resistance mechanism, present in both free-floating and biofilm-associated bacteria, involves the activation of efflux pumps that expel antimicrobial agents. Lastly, antibiotics may become inactivated due to local environmental changes within the biofilm, such as alterations in pH or metal ion concentrations [14]. And lastly, the formation of so called persister cells. Rather than being mutants, these cells are dormant forms of regular cells. They can develop into small colony variants that exhibit high tolerance to external stresses. In the absence of antimicrobial agents persisters which switch back to a normal phenotype can resume growth. Every significant pathogen contains persister cells, which are known to enhance the antibiotic tolerance observed in biofilms and explain the stubborn nature of chronic infections. Furthermore, there are reports that persister cells can play a role in the development of antibiotic resistance [51].

The efficacy of antibiotics against bacterial infections is significantly influenced by the biofilm's developmental stage, with early, reversible phases showing greater susceptibility than more mature formations [44]. Environmental shifts often trigger the transformation from free-floating (planktonic) bacteria to organized biofilms, fostering subsequent adaptive changes [44]. These adaptations can involve alterations in surface molecule expression, virulence factors, and metabolic profiles. For instance, microorganisms might modify the production of capsular polysaccharides and lipopolysaccharides, enhance nutrient acquisition (such as increasing siderophore synthesis for iron recruitment), develop additional antibiotic resistance mechanisms, adjust to varying nitrogen and oxygen levels, or produce toxins like hemolysins and cytotoxic necrotizing factor 1 [10]. Furthermore, certain microbes, like *E. coli* with its N-acetyl-heparosan lyase, possess the capacity to produce enzymes that disrupt and degrade the biofilm matrix. The ability of these microorganisms to ascend against the natural flow in the urinary tract presents a risk of spreading to regions like the bladder and kidneys, and potentially leading to systemic infection [10,35].

Certain microbial species prevalent in urine, such as *Proteus mirabilis*, *Proteus vulgaris*, and *Providencia rettgeri*, exhibit a distinctive metabolic activity: they convert urea into ammonia and carbon dioxide utilizing the enzyme urease [10]. This enzymatic action elevates the local pH, fostering the precipitation of minerals like calcium phosphate and magnesium ammonium phosphate, which can ultimately obstruct urine flow [6]. Within the specific environment of a catheterized urinary tract, *Proteus* species are particularly notable due to two key characteristics: their potent urease production and their remarkable swarming motility across various solid substrates [27]. This includes their capacity to spread effectively over different catheter materials, including those made entirely of silicone, silicone-coated latex, hydrogel-coated latex, and hydrogel/silver-coated latex [27]. Research indicates that, among common urinary tract pathogens, *P. mirabilis* demonstrates superior migratory capabilities across a wide range of standard catheter materials [27].

Beyond well-known pathogens, there's growing evidence suggesting that certain less common microorganisms might contribute to the surprising stability and resilience observed in some biofilms [52,53]. While the specific pathogenic roles of organisms like *D. tsuruhatensis* and *A. xylosoxidans* are still being investigated, they potentially engage in synergistic interactions with *E. coli*. These organisms also appear capable of enduring adverse conditions, adjusting their cell numbers, and modifying their metabolism to optimally benefit from living communally [52,53]. Although the precise mechanisms behind these phenomena are currently unknown, three primary hypotheses have been proposed: the transfer of genetic material from these less common bacteria to *E. coli*; an alteration in the physiological state of susceptible species due to antibiotic absorption; and the enzymatic degradation of antibiotics within the biofilm matrix by enzymes produced by these unusual bacterial inhabitants [10].

In an *in vivo* study by Armbruster et al., specific co-infections of uropathogenic bacteria, namely *P. mirabilis* and *P. stuartii*, were observed to significantly increase the occurrence of complications like urolithiasis and bacteremia [54]. This highlights how understanding biofilm formation can directly inform predictions about a patient's prognosis and clinical outcome, enabling more targeted and effective medication strategies [10].

#### 2.4.4 Microbiological interactions

Within a biofilm environment, the diverse bacterial populations engage in a complex interplay of both antagonistic and agonistic interactions. Antagonistic relationships, where one organism negatively impacts another, can manifest through several distinct mechanisms. These include.

- Resource and Space Competition: Microbes may directly compete for the same ecological niche, or for essential nutrients and physical resources within the biofilm matrix [55].
- Production of Antimicrobial Compounds: Some bacteria synthesize and release toxins, such as bacteriocins, which are detrimental or lethal to other members of the microbial community [56].
- Specialized Secretion Systems: Gram-negative bacteria, for instance, often utilize sophisticated competition mechanisms like the Type VI secretion system (T6SS). This system forms a membrane-puncturing structure, structurally akin to an inverted phage tail and tube, with Hcp and VgrG proteins serving as its crucial components [56].
- Contact-Dependent Growth Inhibition (CDI): Found in both Gram-negative and some Gram-positive bacteria, the CDI system involves a CdiA toxin (secreted by the CdiB outer membrane β-barrel transporter) and a corresponding immunity protein. The N-terminal region of CdiA facilitates adhesion, while its C-terminal domain delivers the toxin into the cytoplasm of a target cell. Notably, CDI systems can operate in ways that are either cooperative or competitive, influencing the establishment of microbial communities and exhibiting a form of kin-selective social behavior [10].

In contrast to antagonistic interactions, synergistic mechanisms highlight how microbial communities can cooperatively enhance each other's survival and growth within a biofilm. These beneficial interdependencies can manifest in several ways.

- Mutual Advantage: Organisms may derive direct benefits from the mere presence of other species, fostering a more robust overall community.
- Enhanced Antimicrobial Resistance: Cooperation among different microbes can lead to a collective increase in the biofilm's overall resistance to antimicrobial agents, surpassing what individual species could achieve.
- Cross-Feeding Dynamics: A common synergistic phenomenon involves the exchange of metabolic byproducts, where one organism's waste product serves as a vital nutrient for another, creating a complex metabolic web.
- Environmental Conditioning: Microorganisms can collectively modify the local environmental conditions within the biofilm, optimizing it to better support the growth and proliferation of their neighboring species [10].

Naturally, an increase in the fitness of one microbial species within a community can sometimes come at the expense of others. This dynamic is often observed with the production of secondary metabolites, frequently referred to as “public goods” [57]. Examples of such molecules include quorum sensing signaling molecules [57], iron-scavenging siderophores [58], and enzymes that degrade antibiotics [10]. Organisms that expend energy to produce these public goods face a challenge from "cheaters" – those that exploit these shared resources without contributing, often leading to the cheaters outcompeting the producers [58]. However, given the widespread presence of polymicrobial biofilms in human infections, it is highly probable that cooperation between different species ultimately strengthens the overall fitness and resistance of the entire biofilm structure [59].

To illustrate this, studies by Azevedo et al. highlight cooperative behavior between *Candida albicans* and *Escherichia coli* in the context of UTIs. Research indicates that *E. coli* enhances *C. albicans*'s capacity to adhere to the bladder mucosa. Furthermore, the presence of *C. albicans* has been shown to boost *Pseudomonas aeruginosa*'s adherence to urinary catheters, while *C. albicans*'s own adherence remains largely unaffected. This latter phenomenon is most likely attributed to the specific attachment of *P. aeruginosa* to the filamentous (hyphal) form of the fungus. It is important to note, however, that while these specific observations were not made in UTI-related settings, *P. aeruginosa* toxins have been observed to exert an antagonistic effect on *C. albicans* within a biofilm [10].

## 3 Treatment

Routine antimicrobial intake is not recommended by SWAB (Dutch Working Party on Antibiotic Policy [Stichting Werkgroep Antibioticabeleid]), IDSA (Infectious Diseases Society of America), European society of Urology (EAU) and Centers for Disease Control and Prevention (CDC) guidelines for prophylaxis in several situations: for patients with either short-term or long-term urinary catheters, for individuals performing intermittent self-catheterization over prolonged durations, or during catheter placement or replacement/removal specifically to prevent CAUTI or CAB. It is also not recommended to screen for bacteriuria in these patients if they are asymptomatic [3,32,60,61]. The recommendation is based on low-quality evidence and a Cochrane review concluded with a statement that one must assess the potential gains from antibiotic prophylaxis against its possible downsides, including the risk of fostering antibiotic-resistant bacteria [3,32,62].

### 3.1 Indication for treatment of CAUTI

Before discussing the treatment of CAUTI it is important to stress the difference between subgroups of catheter associated bacteriuria, CAASB and CAUTI. As already mentioned in the previous chapter (2.1.), CAASB refers to a situation where a single catheter urine specimen shows ≥10^5^ CFU/mL of one or more bacterial species in a patient without any UTI-compatible symptoms; A CAUTI is diagnosed when a patient, either with an indwelling urinary catheter or one removed within the last 48 h, has a single catheter urine specimen containing 10^5^ CFU/mL or more of one or more bacterial species, and the patient has signs or symptoms of UTI with no other identifiable cause [63]. Additionally, pyuria should not be the marker to differentiate between CAASB and CAUTI [63].

When a patient with a newly inserted catheter shows symptoms [72, 73], a CAUTI is indicated once a urine specimen is collected from that catheter. This is crucial given the wide variety of possible infectious agents and the elevated risk of antimicrobial resistance [3]. Treatment in case of CAASB is not recommended since regardless of intake or not of antibiotics symptomatic UTI will occur [64,65]. Furthermore, inappropriate treatment can lead to the emergence of resistant pathogens and to superinfection [66]. The GPIU study on global infection prevalence indicates that the microbes responsible for CAUTI are comparable to those causing other complicated UTIs. This suggests that symptomatic CAUTIs should be treated in line with complicated UTI (cUTI) guidelines [3].

The Loeb criteria, specified for initiation of antimicrobial therapy, consist of symptomatic indicators of CAUTI and at least one of the following conditions should be presented for prescribing an antibiotic therapy: fever, new costovertebral angle tenderness, new onset of delirium, or rigors [66]. It's recommended to hold off on treatment until culture results are available if the patient displays only local symptoms and no signs of systemic infection [60].

### 3.2 Antimicrobial treatment

When a symptomatic CAUTI is diagnosed, you should replace the catheter because of the high likelihood of bacterial biofilm on its surface. After swapping out the catheter, it's vital to collect a urine sample for culture *before* starting any antimicrobial treatment [3, 24]. Additionally, appropriately managing any urological abnormality or the complicating factor causing it is a necessary step. When making decisions, important factors include how severe the illness is at presentation, the prevailing local resistance patterns, and specific patient characteristics such as allergies. According to results from the GPIU studies, the causative pathogens in CAUTI resemble the pathogens in other cUTI [67].

The guidelines for the treatment of complicated UTIs recommend.

- amoxicillin plus an aminoglycoside;
- a second-generation cephalosporin plus an aminoglycoside;
- a third-generation cephalosporin intravenously as empirical treatment of complicated UTI with systemic symptoms [3].

Patients with CAUTI possess an increased risk of having a fluoroquinolone-resistant microorganism. Ciprofloxacin (fluoroquinolones) is recommended only in case when the whole therapy intake is oral, in cases where patients don't need to be hospitalized, or if they experience anaphylaxis to beta-lactam antibiotics and local resistance is under 10 %, this approach is applicable [60]. When fluoroquinolone resistance is observed at a rate of 10 % or higher, the recommended course of action is to administer an initial, single intravenous dose of either a third-generation cephalosporin or an aminoglycoside [60]. Fluoroquinolones are not suitable as empirical treatment for CAUTI patients or when patients had fluoroquinolone intake in the last 6 months, due to high resistant incidence (ciprofloxacin resistant *E. coli* at urology department was described as 25 %, enterobacteria in general had a 11–13 % resistant rate against ciprofloxacin) [60].

For urinary catheters that have been in place for ten days or longer, the suggested treatment involves a combination of co-amoxiclav and gentamicin [60]. It covers *Enterococci* and in case to exclude them, the most adequate treatment option is a 3^rd^generation cephalosporin with gentamicin [60]. In cases where a patient has a history of prolonged indwelling catheterization or relies on intermittent catheterization, an aminoglycoside is often prescribed. This is because such scenarios require coverage against certain less common uropathogens like *Pseudomonas*, *Serratia*, *Providencia*, and *Acinetobacter* [60]. Once the urine culture results become available, the treatment regimen should be adjusted accordingly [60].

### 3.3 Duration of treatment

For individuals experiencing a CAUTI who show rapid symptom resolution, a seven-day course of antimicrobial therapy is generally advised. Conversely, if a patient exhibits a delayed response following antibiotic administration, the recommended treatment duration extends to 10–14 days [3,68]. For patients not experiencing severe illness, a five-day course of levofloxacin may be appropriate [69]. Additionally, a three-day antimicrobial therapy can be considered for women under 65 years old who do not present with upper urinary tract symptoms after their indwelling catheter has been removed [63].

## 4 Urinary catheters

Urinary catheters encompass both indwelling and external types. An indwelling catheter can be inserted either urethrally or suprapubically. The Foley catheter, despite its invention in the 1930s, has remained largely unchanged. Its design flaws, including thick walls, narrow internal diameters, irregular surfaces, and roughly engineered eyelets, have earned it the unflattering title of »healthcare's hidden scandal of neglect « [70]. A safe and reliable system for urine collection and containment is needed, with the specific choice depending on whether the bladder dysfunction involves retention or incontinence. This applies for either short - or long-term use [71]. The most optimal method, for males and females, if feasible, would be clean intermittent self-catheterization, mimicking normal bladder function, while causing minimal physiological and physical damage. If Clean Intermittent Catheterization (CIC) is not compatible with the patients' status, then long term catheterization must be used [71].

Urethral catheterization is indicated in several clinical scenarios, including but not limited to Ref. [67].

- Urinary Retention: Addressing both acute and chronic inability to empty the bladder.
- Voiding Dysfunction: Managing difficulties with urination, particularly those stemming from neurological conditions or obstructions at the bladder outlet.
- Monitoring Urine Production: Precisely measuring urinary output, especially during surgical procedures or in critically ill individuals.
- Intravesical Treatments: Facilitating direct bladder therapies such as irrigation or lavage.
- Surgical Procedures: As a supportive measure during various surgical interventions.
- Incontinence Management: Aiding in the healing of open sacral or perineal wounds, preserving skin integrity, or providing comfort for intractable incontinence.
- Extended Immobilization: When a patient is expected to be immobile for a prolonged period.
- Bladder Decompression: Relieving pressure within the bladder.
- Enhanced Comfort in Palliative Care: Improving patient comfort during end-of-life care.

Urinary retention as well as voiding dysfunction often requires long-term catheter use. Similarly, the issue of catheter overuse, particularly for indwelling urethral catheters, stems from their application in comparable scenarios [72]. These include acute urinary retention or bladder outlet obstruction, the need for precise urinary output measurements in critically ill patients, perioperative use for specific surgical procedures, aiding in the healing of open sacral or perineal wounds in incontinent individuals, managing immobilization, and enhancing comfort in palliative care settings. It's particularly important to note that urinary catheters are generally best avoided for incontinence management in both hospital patients and those residing in nursing homes [72].

A significant hurdle in the medical device market involves developing products that offer an optimal balance of price and performance, particularly without undermining the body's inherent defenses against infection [17].

An ideal catheter system should incorporate several key features.

- Ease of Use and Minimal Friction: It should allow for simple removal and insertion, with minimal friction against the urethra [71].
- Effective Bladder Drainage: The catheter's retention system must facilitate complete bladder filling and emptying, ensuring no residual urine remains [17].
- Physiological Mimicry: While in place, the catheter should permit the bladder to fill at low pressure and empty entirely, closely mimicking the organ's natural physiology [71].
- Airlock Prevention: The design, perhaps through novel tubing, should prevent the formation of airlocks [17,39].
- Blockage Prevention: To counter bacterial encrustation, the system should incorporate measures like precautionary sensors that change color in the presence of *Proteus mirabilis* [73], special antibacterial coatings, or the use of new materials [14,17,19].
- Safe Suprapubic Insertion: It must allow for safe insertion via the suprapubic route [71].
- Accessible Control: The control mechanism should be manageable for all users, including those with impaired cognitive or manual abilities [71].
- Biocompatibility: The materials used must be compatible with the body to prevent adverse reactions [17].

Bladder drainage can be achieved in two main ways: either by following the natural path through the urethra (transurethral) or by creating a new, artificial opening (suprapubic) that connects the lower abdominal wall directly to the bladder [71]. The female urethra is typically short, muscular, and straight, measuring around 40 mm. In contrast, the male urethra is considerably longer, about 160 mm, more sensitive, and features two anatomical curves. This is why some catheters have a curved shape (*coudé and bicoudé*) to minimize the risk of iatrogenic urethral trauma with proper use [71]. Suprapubic catheterization is associated with more complications in unexperienced hands, due to the danger of perforating the bowel or causing haemorrhaging [74]. On the other hand, a prospective cohort study revealed that patients with suprapubic catheters experienced fewer catheter-associated UTIs (CAUTIs) and had shorter hospital stays, though they were more prone to colonization by multi-drug resistant organisms, thus could be the better option for long-term catheterization [75].

A catheter, essentially a hollow polymeric tube, is carefully advanced through the urethra. This tube incorporates an inflation channel, allowing a syringe to inflate a balloon at its tip with sterile water [71]. To secure the catheter within the bladder, its smooth, rounded end extends beyond this balloon. Near this tip, one or more small openings, or eye-holes, are cut into the tube to facilitate urine drainage [76]. Some catheter designs include a third channel, which can be utilized to infuse saline or other irrigating fluid into the bladder, particularly in situations where blood clot formation is a concern, such as in a postoperative setting [71].

Certain catheters available today feature two balloons at their distal end; the additional balloon, positioned at the very tip, is intended to lessen the chance of urothelial trauma. Drainage occurs through small perforations located between these two balloons, with the more proximal balloon serving as the retention mechanism. A potential drawback of this dual-balloon design, however, is the increased risk of residual urine remaining in the bladder after drainage, which could consequently elevate the risk of catheter-associated urinary tract infections (CAUTI) [71].

Catheter dimensions are typically indicated in Charriere (Ch) units, where 1 Ch corresponds to a 0.33 mm diameter, or in French gauge (Fr or FG), which represents the circumference in millimeters. Standard clinical practice generally recommends using the smallest catheter bore that still allows for effective drainage [71]. Sizes most commonly employed range from 12 to 16 Fr. To reduce the likelihood of catheter obstruction, particularly in the presence of infection or anticipated postoperative bleeding, a larger bore catheter is often selected. The majority of urethral catheters manufactured range in length from 41 to 45 cm [77].

For men experiencing urinary incontinence, an external or condom catheter can be a practical option for both short-term and long-term use. This system involves a sheath that fits over the penis, connected via a tube to a collection bag that can be worn on the leg and emptied periodically.

While seemingly appealing, this method comes with several notable drawbacks. Up to 40 % of users may develop urinary tract infections, and 15 % experience complications like inflammation, ulceration, necrosis, gangrene, or constriction of the penile skin. There's also the constant risk of the condom detaching and urine leaking [71]. Furthermore, caring for a condom catheter demands a significant amount of nursing time. Although not typically the primary treatment for male urinary incontinence, the condom catheter does prove quite useful for non-invasively assessing bladder pressure [71].

### 4.1 Short history of catheters development

Urinary catheters date almost 3500 years back [71]. The term "catheter" originates from the ancient Greek word "kathiénai", which directly translates to "to thrust into" or "to send down" [77]. Prior to the 1930s, before the widespread adoption of the familiar Foley catheter, the practice of catheterization was almost entirely confined to managing urinary retention in men. This was largely due to the relatively infrequent occurrence of this condition in women [71]. The early catheters were usually rigid and designed for intermittent catheterization [4].

The evolution of catheters has seen the incorporation of a diverse array of materials since their earliest conceptualizations. Historical records indicate that some of the very first documented materials used include copper, tin, bronze, and gold by the Greek physiologist Erasistratus, while the Egyptians utilized lead and papyrus. In China around 100 B.C. [4], lacquered or oiled onion stems, dried reeds, and palm leaves served this purpose. A significant advancement occurred in 1779 when French jeweler and goldsmith Bernard crafted the first flexible, gum-elastic catheter [4]. This design was further refined in 1853 with the addition of a retention balloon, fashioned from rubber or woven fabric that had been dipped in linseed oil and subsequently baked [4].

The widely recognized Foley catheter made its debut in 1936, initially constructed from latex [76]. Its distinctive shape is achieved through a manufacturing process that involves immersing a catheter former in a coagulant before dipping it into a latex mixture. The final thickness of the device is built up through repeated dippings, with the viscosity of the latex and the speed at which the former is withdrawn significantly influencing the end product. The ultimate step in this process is vulcanization, which imparts the characteristic rubber properties of resilience and strength to the catheter. Though initially intended for post-prostatectomy hemostasis, its application quickly broadened to become commonplace in managing urinary incontinence and retention [4]. However, latex, due to its inherent cytotoxicity, frequently led to urethritis and urethral strictures, and instances of encrustation and infections were almost inevitable with prolonged catheterization [71]. Modern latex catheters are now commonly coated with silicone elastomer to mitigate these issues. Furthermore, latex's capacity to absorb up to 40 % of its own weight in water could cause its external diameter to increase, thereby reducing the internal lumen size. This characteristic generally limited the useable lifespan of latex devices to approximately 14 days [4].

These limitations prompted the introduction of new silicone catheters in 1968. Silicone offers distinct advantages, being non-allergenic and superior to latex catheters in terms of both kink resistance and fluid flow [4]. By 2001, catheters incorporating chemical impregnation and antimicrobial coatings had been introduced. To this day, the urethral catheter (UC) remains the most commonly utilized device for patients experiencing urinary incontinence (UI) [71]. Further catheter types, designs, and specific characteristics will be explored in subsequent sections.

#### 4.1.1 Catheter materials

EAU in their guidelines describes following available catheter materials.

- Polyvinyl chloride (PVC) stands as the most prevalent material for catheters, valued for its transparency, flexibility, and sufficient firmness that facilitates straightforward insertion [67,78]. At body temperature, it warms a bit, and the catheter can be more pliable. PVC catheters were considered as standard in patient's tolerability, but due to the environmental awareness today, PVC-free catheters are preferred [79].
- Silicone is a biocompatible synthetic material that has low toxicity and tissue inflammation rate. It also has the right properties for designing a flexible and thin-walled catheter [67,78].
- Ethylene vinyl acetate (EVA) is an environmentally friendly polymer material as it does not contain phthalates. Furthermore, it is soft, flexible as well as stress-crack resistant, resistant to UV radiation and waterproof [67,78,80].
- Coated catheters have hydrophilic coating, mostly polyvinyl-pyrrolidone (PVP), while sodium is used providing smoother surface when wet and thus minimizing the risk for urethral trauma [81]. Another type of coating is antibiotic impregnation e.g., nitrofurazone used for a local treatment and at the same time minimizing superinfection risk [81]. Healthcare Infection Control Practices Advisory Committee (HICPAC)for CDC suggest preferable use of hydrophilic catheters [72].
- A Closed-Catheter system is used for sterile or aseptic techniques and offers a pre-lubricated product with an already integrated collection bag [81].
- Uncoated catheters are used for clean techniques and are made of red rubber latex, which is very flexible and therefore sometimes difficult to insert, or are latex-free, which are the most commonly used and made of PVC or silicone [81].
- While latex is an affordable and pliable material, its surface presents notable drawbacks. Its high friction can lead to increased vulnerability and rapid encrustation from urine mineral deposits. Furthermore, latex carries the risk of inducing allergic reactions and exerting a cytotoxic effect on mucosal tissues, which can result in inflammation and urethral strictures, particularly with prolonged catheterization [[82], [83], [84]]. Latex has a tendency to absorb bodily fluids, which can lead to a reduction in the catheter's internal drainage lumen and an increase in its outer diameter [85]. Several coatings have been developed in recent years, to improve clinical characteristics of the latex: silver, hydrogels, polytetrafluoroethylene (PTF, Teflon) and silicone coatings [86].
- Silicone catheters, designed as thin-walled catheters, are hypoallergenic, with a larger lumen that might have an impact to delayed encrustation and catheter blockage [85]. They can also be. are desirable material [86]. On the other side, in 1983 it was found out that the Foley catheters' (manufactured by Dover and Travenol) silicone bulb filled with sterile water had a marked amount of fluid, which increases the risk of displacement [87]. A drawback of silicone catheters is their increased tendency to develop a cuff upon deflation, potentially causing discomfort during removal or injury to the urethra [85].
- Despite the long history of designing the urinary catheters, there is currently still no synthetic biomaterial on the market without any effect on the tissue as well without promoting anyhow biofilm formation. There is also a lack of clear evidence for defining the best type of indwelling catheter [88,89]. Both the CDC and EAU continue to endorse (and preferably suggest) silicone catheters, as they are associated with less encrustation in individuals requiring long-term catheterization [72,[90], [91], [92]].

A study conducted by Kumon et al. evaluated bacterial attachment scores across different catheter materials: rubber (which closely resembles latex), silicone, and hydrogel-coated latex. Their findings indicated that silicone exhibited the lowest average bacterial attachment, scoring 1.3. Hydrogel-coated latex showed a moderate score of 2.3, while rubber had the highest average attachment at 3.6. (These scores were based on a scale where 1 denoted no bacterial attachment, 2 indicated scattered attachment, 3 represented uniform attachment without biofilm, 4 signified mild biofilm formation, and 5 denoted strong biofilm formation). This consistent pattern, demonstrating silicone's superior resistance to bacterial adherence, was observed across a wide range of common urinary tract pathogens, with the sole exception being *Pseudomonas aeruginosa* [93].

Worth noting is that hydrogel-coated catheters with water absorption increase lubriciousness outside the surface and have demonstrated the ability to reduce the cytotoxicity shown in the past [86]. Teflon-coated latex catheters are smoother as plain latex catheters and are designed to reduce friction. Due to the hydrophobic characteristic of Teflon, they repel water and so minimize the swelling of catheter [86]. Because of this latex catheters are being suggested to be avoided if possible or in use for a short-term period [90].

### 4.2 Intermittent urinary catheter

The term clean intermittent catheterization (CIC) represents the action of insertion and removal of a catheter several times daily with the purpose to complete emptying the urinary bladder for a relief of urinary retention due to idiopathic or neurogenic dysfunction. It also allows normal bladder dynamics [81]. For long-term management of a neurogenic bladder, intermittent self-catheterization is the preferred method, carrying a lower risk of UTI compared to indwelling catheters [72,81]. Other risk factors for developing a UTI when using IC beside virulence factors of bacteria are a bad catheterization technique and passing the catheter trough the very contaminated area of the urethra, especially distal urethra (*E. coli)* [81,94].

#### 4.2.1 Design and types

Itermittent catheters (ICs) are designed with gender-specific considerations and come in a range of sizes, as previously noted. Sizes from 6 to 12 Fr are typically for pediatric use, while adult catheters generally range from 14 to 22 Fr. The catheter tip itself can either be straight, commonly tapered to facilitate smooth insertion, or curved, which aids easier navigation in narrower urethras [81]. Catheters are packed in a sterile packing and in different European countries there are different policies about reimbursement and catheter reuse which. This could be one of the reasons for increased risk of complication in catheterization [78]. The One-way or straight catheter has in cross-section only one lumen and does not have a balloon on the top as it is not designed to remain in the bladder for a longer period of time. This type of catheter is used for intermittent catheterization, treating urethral strictures as well as instillation of drugs in the bladder, for urodynamic investigations and for suprapubic catheterization without balloon [90].

### 4.3 Indwelling urinary catheters

Indwelling catheters are made to hold a position in the urethra and compared to intermittent catheterization patients, causea higher risk for developing UTI [72,81]. There are two primary methods for indwelling catheterization: transurethral catheterization and suprapubic catheterization. Transurethral catheterization follows the natural route through the urethra, much like intermittent catheterization. In contrast, suprapubic catheterization involves inserting the catheter directly into the bladder through the anterior abdominal wall.

#### 4.3.1 Desing and types

The Two-way or so-called Foley catheter has in cross-section two lumens, one for urine drainage and another for filling the balloon at the top of catheter. This type of catheter is the most commonly used of all types [90].

The Three-way catheter is distinguished by having three distinct lumens in its cross-section, with the third channel specifically designed to enable continuous bladder irrigation. This particular type of catheter is typically utilized following urological surgery or whenever the bladder requires ongoing or intermittent irrigation. A common application, for instance, is when bleeding from the bladder or a tumor results in blood clots or debris that need to be flushed out [90].

A catheter with integrated temperature sensor is designed to measure “deep” body temperature when needed [90,95,96]. A Suprapubic catheter as an alternative to urethral catheterization. Again, different models exist: The Foley balloon catheter, like Foley urethral catheter; catheter without a balloon and the third model is a Foley balloon catheter with an open end. Suprapubic type of catheterization is believed to carry less risk for catheter-induced urethritis and catheter contamination with bowel micro-organism [90].

### 4.4 External urinary catheters

External catheters (ECs) are primarily made as a relatively non-invasive utility for male urinary incontinence as an alternative for invasive catheterization or absorbent pads. Mixed study results are found comparing EC versus indwelling catheter. They estimated that 40 % of men using external catheters developed UTIs, and notably, the average microbial count in these cases was considerably higher than in those using indwelling catheters [97].

The limitations of current reactive treatments, exacerbated by biofilm‐mediated resistance, underscore the need for proactive, materials‐based interventions to prevent CAUTI before they occur—strategies explored in the following section.

## 5 (PREVENTION) strategies to combat urinary tract infections

The need for preventive strategies and novel approaches has been requested and mentioned many times [7,9,14,17,19,70,71]. Strategies to tackle this problem range from human vigilance and hygiene to novel, smart and multi-functional coated catheters [9]. Naturally, rigorous hand hygiene is recommended to prevent all infections acquired in healthcare settings [9,34]. Chenoweth et al. have identified that the majority of outbreaks involving urinary pathogens are linked to inadequate hand hygiene practices among healthcare staff [9]. Another well-known, yet not always heeded enough, preventive measure is the reduction of usage of broad-spectrum antibiotics. This will help prevent the development of antimicrobial resistance related to urinary catheters [9]. Furthermore, the adherence to clinical guidelines, indicated and appropriate use of catheters, a monitoring system (computerized or human - e.g., nurse-based reminder) for catheterization have shown promising results [18]. Naturally, effective strategies for prevention include using a closed drainage system and promptly removing the catheter [35]. Maintaining a closed drainage system, where the collection tube is securely sealed to the drainage bag, substantially lowers the occurrence of bacteriuria. For male patients, adopting this method cuts the incidence of bacteriuria from 95 % after 96 h of open drainage down to 50 % after 14 days. Similarly, for women, it reduces the rate to 50 % after 11 days of closed drainage [38].

**Fig. 6:**
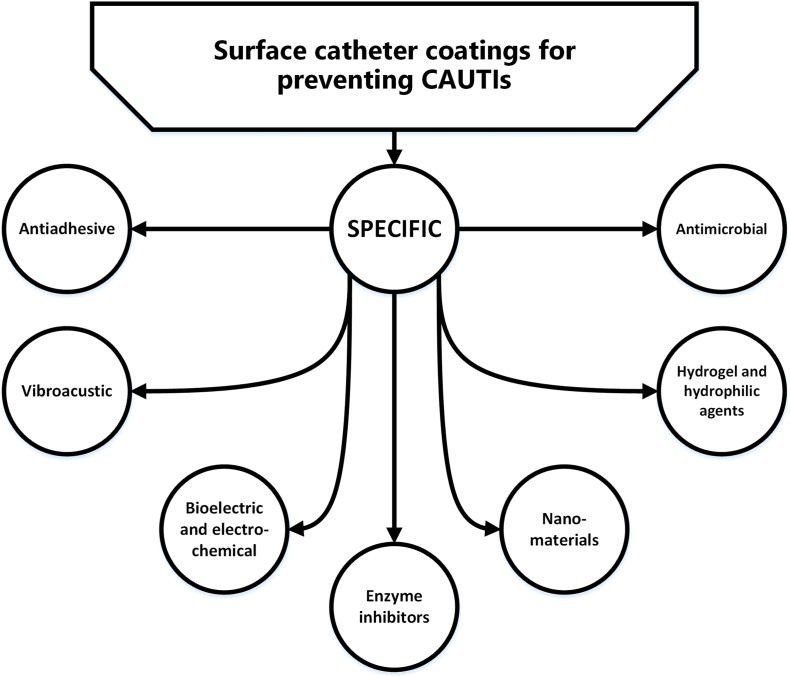
Potential strategies for catheter development

Source: own.

Preventative strategies can generally be categorized into two main approaches. The first focuses on altering the surface of the base catheter (bio)material itself. This often involves introducing highly specific topographic features at submicrometric or nanometric scales. By doing so, the contact area between bacterial cells and the surface is reduced, making microbial attachment less favorable [98,99]. A frequently used concept is manipulating the surface hydrophilicity [19,35,100]. The second approach focuses on the development of specific antimicrobial coatings, e.g., using nitrofurazone, minocycline, or rifampicin, while also using other agents to build desirable “bioactive” catheters [19,35]. Other potential approaches that include new technologies are depicted in Fig. 6, are further discussed below. A summary of important studies is presented in Table S1.

### 5.1 Surface-engineered solutions

#### 5.1.1 Antiadhesive coatings

Inspired by naturally occurring topographical features on surfaces, precise surface patterns are engineered to study cell-surface interactions in the development of biofilms. Shark skin, lotus leaves, or butterfly wing patterns are often mimicked on man-made materials to reduce biofilm formation as they exhibit specific pattern shapes and spatial distribution [101]. Some of these patterned surfaces are already trademarked and commercially available, like Sharklet AF™ [102], but have so far not been extensively tested against *P. mirabilis* and exposed to *in vivo* tests on urethral catheters [103].

A minimum of three surface topography features were identified as necessary to adequately characterise surface topographical features in cell adhesion studies; summit density, developed area ratio, and root-mean-square surface roughness [101]. Chung et al. spatially patterned silicone (Sharklet AF ™) and exposed it to *E. coli* for 21 days, which resulted in a decrease in surface coverage by over 40 % in tryptic soy broth media [102]. Perera-Costa et al. engineered various surface topographies on silicone by varying the height/depth and characteristic length of the patterns, their interstitial space, and the average surface roughness. The overall conclusion was that the patterned surfaces reduced biofilm density of *S. epidermidis*, *Bacillus subtilis*, and *E. coli* by up to 45 % when compared to smooth silicone regardless of the surface hydrophilicity/hydrophobicity [104]. Mon et al. manufactured colloidal crystal monolayers on silicone which reduced the density of colony-forming units of *P. aeruginosa* by 99 % increasing it to 99.9 % with the addition of antibiotics [105]. Even though the influence of antibiotics was minimal and surface topography was the predominant factor, it seems that additional surface modification is beneficiary when considering use in biomedical applications [105]. In this regard, S-nitroso-N-acetylpenicillamine doped submicron patterned polyurethane films showed a reduction of *S. epidermidis* cells of up to 88 % when compared to smooth-coated films as shown by Xu et al. [106]. Despite the fact that great advances were made, different types of bacteria attach and grow with different dynamics on surfaces with varying topographical features as well as with various chemical compositions, thus making it challenging for scientists to engineer patterned surfaces that reduce biofilm development and growth of a broad bacterial spectrum [107].

A meta-analysis published in 2024 confirms that micro/nanopatterned silicone (e.g., Sharklet™, colloidal-crystal arrays) still achieves the largest physical-only biofilm reduction, but stresses that pairing low-surface-energy chemistries with topography is essential to curb *P. mirabilis*-driven encrustation [108]. Complementing topography, Cai et al. grafted zwitterionic RAFT-polymer brushes onto polyurethane and recorded a successful suppression of *E. coli*/*P. aeruginosa* under 72 h artificial-urine flow without cytotoxicity [109].

#### 5.1.2 Vibroacustic coatings

Surface acoustic waves (SAW) have demonstrated an ability to disrupt the adhesion of free-floating microorganisms in various environments, from food processing to water decontamination [[110], [111], [112]]. Catheters equipped with piezo elements can generate low-energy acoustic waves that travel through the device, producing a vibrating layer. This vibration then extends into the surrounding fluid, causing bacteria to vibrate and thereby impeding their attachment. Currently, the *in vivo* application of therapeutic ultrasound (US) for antimicrobial treatment primarily leverages its capacity to boost antibiotic effectiveness. For example, this method is employed to eradicate infections in joint prostheses by combining gentamicin with ultrasound activity [110,113]. Research conducted by Kim and colleagues explored a novel approach combining a surface acoustic wave biofilm sensor with a therapeutic method based on the bioelectric effect. This integrated system, as they described, holds the potential to not only identify biofilm formation in its early stages but also to actively treat it [114]. Bandara et al. used low-frequency vibrations in combination with tobramycin to kill *P. aeruginosa* biofilms at sub-MIC [115]. Nonetheless, a phenomenon has been observed where some bacteria (e.g. *P. aeruginosa)* react by producing a more potent biofilm, demonstrating significant resistance to subsequent therapeutic efforts*)* [110]. Advancing the application of this method will necessitate a precise understanding of how ultrasound activity influences bacterial quorum sensing and gene expression. To conclude, low-frequency (0.8 kHz) surface-acoustic-wave stimulation delays *Proteus* crystal nucleation by ≈ 24 h in dynamic bladder models and potentiates sub-MIC tobramycin by ∼2-fold. RNA-seq showed down-regulation of quorum-sensing loci but inducible EPS genes, highlighting the need for precise dose mapping [116].

#### 5.1.3 Hydrogels and hydrophilic coatings

Composed of cross-linked, insoluble, and hydrophilic polymers, hydrogels are characterized by their ability to trap water [14]. Such coatings are designed to provide improved patient comfort, decrease microbial adherence, and reduce encrustation [7]. Although, catheters coated with hydrogel are more comfortable for the patients, their ability to prevent infections and biofilm formation, still remains controversial [7,14]. Observations indicated that the presence of a hydrogel layer promoted the aggregation of free-floating cells. This aggregation, in turn, led to an increased formation of crystal nuclei, ultimately causing more rapid catheter obstruction compared to uncoated silicone [117]. In an in vitro urinary model [7], hydrogel-coated latex catheters became obstructed faster (at 34 h) than either uncoated silicone (at 48 h) or silicone-coated latex (at 38 h). Other researchers have noted the advantages of hydrophilic catheters over PVC catheters, including fewer urinary tract infections, less microhematuria, and higher patient satisfaction [118,119]. Nevertheless, the drawbacks of hydrophilic hydrogel-based coatings must be taken into account and evidence suggests that adding active agents to the hydrogel can improve the catheters performance when dealing with biofilm growth [117]. Even though progress is being made, most of the commercially available hydrogel coatings with active ingredients still fail to prevent biofilm encrustation of catheters, especially crystalline encrustation driven by *P. mirabilis* [103]. Extensive research is still focused on designing new and improved hydrophilic or hydrogel-based coatings with specific active ingredients and their combinations, which would be able to tackle the complicated processes of biofilm formation [119]. For example, a double-network PVA/chitosan hydrogel doped with Ag quantum dots kept *P. mirabilis* colonization below detection for seven days and prevented catheter blockage in a dynamic encrustation loop while preserving 95 % urothelial-cell viability [120]. Furthermore, colour-change pH sensors have now been co-grafted into such hydrogels, enabling bedside detection of early crystalline biofilm formation [73].

Naturally occurring charged molecules like polysaccharides and peptides are gaining great interest when developing new responsive hydrophilic coatings [121,122]. Highly negatively charged polysaccharide coatings can reduce the adhesion of cationic biomolecules like proteins and reduce the deposition and growth of *S. aureus* pathogen on polydimethylsiloxane silicone [121]. Zwiterionic molecules and oppositely charged multilayer coatings are being studied as well as they combine both antiadhesive and antimicrobial properties [123,124]. Zwitterionic carboxymethyl chitosan showed to reduce static biofilm growth of *E. Coli* and *P. mirabilis* but did not reduce biofilm growth of the latter under dynamic conditions due to the high cell motility of *P. mirabilis*, which interestingly increased with increasing hydrophilicity of the coating [124]. Curd-peptide base hydrogels, formulated from a mixture of probiotic bacteria including *Streptococcus thermophilus*, *Lactobacillus casei*, and *Bifidobacterium bifidus*, have demonstrated effectiveness in curbing the non-crystalline biofilm growth of *Staphylococcus aureus* and *Pseudomonas aeruginosa*. This reduction is attributed to a multifaceted action, combining the hydrogel's inherent water content with its antimicrobial properties and its capacity to inhibit bacterial quorum sensing [122]. When it comes to molecules that directly affect bacterial behaviour and biofilm formation, various antimicrobial agents and other chemical compounds have been applied as catheter coatings. In catheters coated with hydrogel-silver alloy coating a decrease of up to 45 % of CAUTI has been observed [14]. A coating combining minocycline and rifampicin has demonstrated effectiveness in preventing biofilm formation by both Gram-positive and Gram-negative pathogens, with the notable exceptions of *P. aeruginosa* and *Candida* species [125]. With the use of such coating one must keep in mind the possibility of the development of resistant phenotypes amongst bacteria. Another recent study has compared the rate of UTIs associated with hydrophilic-coated catheters versus uncoated PVC catheters [126]. The results indicated a beneficial effect regarding clinical UTI when using hydrophilic-coated catheters in terms of fewer cases of symptomatic UTI.

In summary, while anti-adhesion coatings demonstrate consistent in vitro biofilm reduction, clinical translation remains limited by durability under dynamic urinary conditions, manufacturing scalability, and variable efficacy against crystalline biofilms. Addressing these issues will be critical for broader adoption.

#### 5.1.4 Antimicrobial coatings/contact-active coatings

Polymers used for antimicrobial coatings on urinary catheters function either as carriers for other types of antimicrobial agents including biocide-releasing chemicals (such as silver ions or nitric oxide) [127] or they have antimicrobial function by themselves [128].

Known since antiquity, silver's ability to kill bacteria has led to its wide range of applications [129,130]. The bactericidal effects of silver ions stem from several actions, including their ability to inactivate critical enzymes by binding with thiol groups, enhance pyrimidine dimerization through photodynamic reactions, and alter cell walls due to electron-dense granules [7,131]. According to findings by Desai et al., the observed decrease in asymptomatic bacteriuria when using silver-hydrogel-latex catheters might be primarily attributable to the hydrogel coating itself, rather than the silver. Their experimental setup indicated that silver impregnation had only a negligible impact on bacterial adherence [132]. In their International Clinical Practice Guidelines, Hooton et al. reported that silver oxide catheters, which are no longer commercially available, did not demonstrate a statistically significant decrease in catheter-associated bacteriuria [61]. Conversely, silver alloy catheters were observed to significantly lower the rate of catheter-associated asymptomatic bacteriuria (CA-ASB) in hospitalized adults who were catheterized for one week [61]. Additionally, other meta-analyses examining antimicrobial catheter trials have found that silver oxide-coated catheters are ineffective, but silver alloy-coated catheters provide a protective effect against CA-bacteriuria [61]. Regev-Shoshani et al. describe the efficacy of silver alloy-coated catheters appear to be considerably lower in current research when contrasted with previous findings. In their investigation, a silver-alloy coating was indeed found to halve the growth of bacteria embedded within biofilms [133]. Furthermore, a comprehensive meta-analysis indicated that silver alloy catheters significantly reduced asymptomatic bacteriuria in adult patients catheterized for under a week; however, this benefit diminished in those catheterized for more than seven days [134]. Ultimately, while anti-infective urinary catheters appear to reduce bacteriuria in individuals undergoing short-term catheterization, there remains a lack of conclusive evidence that these devices effectively prevent catheter-associated urinary tract infections (CAUTI), bloodstream infections linked to UTIs, or fatalities. Consequently, anti-infective urinary catheters are not routinely advised for CAUTI prevention [9]. An interesting approach was reported by Won et al., who created a dual-layer nano-engineered Foley catheter in which a ZnO–Ag topcoat was positioned on a nitric-oxide-releasing polyurethane reservoir. Their construct achieved a high reduction of polymicrobial biofilms in a porcine model with no increase in inflammation [135]. Separately, Ding et al. reported copper-phenolic metal-organic networks that release Cu^2+^ at only 0.07 μg cm^−2^ d^−1^ yet killed *S. aureus* and *E. coli* within 2 h accompanied with a negligible haemolysis [136].

Other contact-active antibacterial agents include cationic polymers, such as quaternary ammonium compounds. These compounds are effective against a broad range of Gram-positive and Gram-negative bacteria. Their efficacy stems from their cationic charge and other functional groups, which disrupt essential intermolecular interactions within bacterial enzymes and membrane components [[137], [138], [139], [140]]. Considerable research has already focused on chitosan and its various derivatives, a notable category of natural cationic polymers. Chitosan itself is produced through the partial deacetylation of chitin, which is a naturally abundant and renewable resource. This process yields a family of polysaccharides composed of β-[1–4] linked units of both glucosamine (2-amino-2-deoxy-D-glucose) and N-acetyl glucosamine (2-acetamido-2-deoxy-D-glucose) [141]. At low pH, amino groups, present in the polymer structure, allow interactions with negatively charged [141,142] compounds resulting in antibacterial activity. Research conducted by Campana et al. [141] demonstrated a notable reduction in biofilm formation on urinary catheters when various molecular weights of chitosan were introduced into an artificial urine medium (AUM). The authors specifically observed that the inclusion of chitosans in the medium significantly hindered the ability of both *K. pneumoniae* and *E. coli* strains to form biofilms. Given its low toxicity to mammalian cells and good biodegradability, chitosan presents itself as a promising candidate compound for future non-antibiotic coatings aimed at preventing catheter-associated urinary tract infections (CAUTIs) [141].

The other group of polymer-based antimicrobial coatings acting as antibiotic/biocidal or drug-releasing agents [128] are.

- incorporated throughout the volume of polymer matrix or
- immobilized as coatings on the polymer surface [128].

In order to prevent encrustation and resist bacterial adhesion, Dayyoub et al. [143] have developed a poly(lactic-co-glycolic acid) (PLGA) film possessing both antibacterial and anti-encrustation properties. This film was engineered to incorporate two distinct antibacterial agents: norfloxacin and tetraether lipid-coated silver nanoparticles. When tested against uncoated sheets, these films effectively inhibited bacterial adhesion in laboratory settings and even reduced the viability of bacteria on the surface. For the encrustation experiments, the researchers observed that the acidic byproducts resulting from PLGA degradation successfully neutralized the alkaline substances produced by urea hydrolysis [143]. Sileika et al. [147] also demonstrated a coating strategy for surfaces that both deters bacterial attachment and actively eradicates bacteria via silver release. In this instance, a biopolymer called polydopamine served as a "primer." An antifouling polymer (PEG) was then grafted onto it, and silver nanoparticles were nucleated into it from a silver salt solution. This system turned out to have very efficient antifouling (due to PEG) and antimicrobial (due to silver NPs) performance against both gram-positive and gram-negative bacterial strains.

#### 5.1.5 Coatings wit bioelectric and electrochemical effects

Managing biofilm-related infections often involves the use of antibiotics or biocides, but these approaches present several significant challenges [144,145]. It's tough to ensure a steady, continuous delivery of these agents to the infection site. Furthermore, biofilms act as a diffusion barrier, making it difficult to control the concentration of antibiotics or biocides directly at the surface where they're needed most. A significant concern is also the potential for biofilm cells to develop resistance, which can render treatments less effective over time. Lastly, there are instances where biocides react with existing biofouling deposits, inadvertently impeding their own diffusion and effectiveness [144,145].

Iontophoresis describes a physical process where ions are compelled to move diffusively through a medium by means of an applied electric field [14]. Laboratory studies have demonstrated its capacity to boost the effectiveness of antibiotics against bacteria embedded within biofilms [144]. Raad et al. reported in their study that an iontophoretic catheter utilizing silver, is known for its broad-spectrum bacterial inhibition, extended durability, and high efficacy in preventing colonization *in vivo* [146]. A lot of research has shown that using a direct electric current significantly boosts how well antimicrobial agents work in lab tests. This effect is known as the bioelectric effect [144,147].

Electrochemical biofilm control is a technology that modifies surface characteristics or triggers reactions to either prevent or stop cell attachment, or to detach existing cells from a surface [147]. While not yet universally confirmed, the proposed ways this technology works include.

- Generating Strong Oxidants: Electrochemical reactions can produce powerful oxidants, such as hydrogen peroxide, which forms from the partial reduction of oxygen on metal surfaces [144,145].
- Ion Migration and Oxygen Generation: Water electrolysis can cause ions to migrate and generate oxygen [144].
- Electrostatic or Electrophoretic Repulsive Forces: The technology can also create repulsive forces that push cells away, either through electrostatic interactions or electrophoresis [144].

Newer approaches indicate that applying an electrical current can enhance the efficacy of antibiotics and biocides [144,[147], [148], [149]]. However, Sultana et al. have specifically stated that for this technology to be used in clinical settings, it's crucial to understand how it works and confirm its effectiveness across different types of antimicrobial agents [147]. Especially because the literature regarding this topic is conflicting [147]. The reason as to why, can be attributed to the lack of both current or potential data and geometric description as well as it is important to distinguish between electric field-induced and electrochemically controlled systems for biofilm control when evaluating experimental data, as they are often conflated [147].

#### 5.1.6 Enzyme inhibiting coatings

In the ongoing search for novel antibiofilm compounds, the urease enzyme, particularly from *Proteus mirabilis*, has become a significant research target. Studies by Abdullah et al. [150], demonstrated that both levofloxacin and ciprofloxacin displayed excellent inhibitory activity against this urease enzyme, alongside remarkable effectiveness against *Proteus mirabilis* and *Helicobacter pylori*. This same beneficial effect was also observed with a ciprofloxacin hydrazide derivative and levofloxacin hydroxamic acid [151]. N-acetyl-d-glucosamine-1-phosphate acetyltransferase is crucial for the biosynthesis of peptidoglycans, lipopolysaccharides and adhesins in certain bacteria. Certain inhibitors of this enzyme have shown good antibiofilm activity [152]. Abdel-Baky et al. evaluated the effect of *N*-acetyl cysteine and dipropyl disulphide and concluded that they could be used in the control of *P. mirabilis* urinary tract infections [153]. Natural compounds as vanillic acid [154], allicin from garlic [155], *Rhizoma Coptidis* protoberberine alkaloids [156] and natural plum juice [157] have been tested and yielded promising results.

#### 5.1.7 Bacteriophages-based coatings

Viruses known as bacteriophages (or phages) selectively infect bacteria. Lytic phages are particularly noteworthy as they interrupt bacterial metabolism for their own replication, leading to rapid lysis of the host bacterium [158]. Bacteriophages are very specific, meaning they're less likely to disturb the normal microbial balance than current antibiotics. Although resistance has been observed, a "phage cocktail" can help avoid this issue. [158]. Phages have also been incorporated into hydrogel-coating catheters. The combination resulted in a reduction in biofilm formation by *S. epidermidis* and *P. aeruginosa* [159]. Phage cocktails embedded in PEG hydrogels now remain lytic for ≥10 days at 37 °C and delay mixed *E. coli/Candida* biofilms on latex by 48 h.

#### 5.1.8 Quorum sensing inhibitors coatings

Quorum sensing (QS) is a sophisticated chemical signaling system that enables bacteria to make collective decisions based on their population density. This mechanism allows them to regulate crucial processes, including bacterial biofilm formation and the expression of virulence factors such as motility. The communication occurs through small signal molecules known as autoinducers. These include fatty acid derivatives like N-acylhomoserine lactones (AHLs), commonly employed by Gram-negative bacteria, and autoinducer-2 (AI-2), a furanosyl borate diester vital for interspecies communication [160]. Other identified signaling molecules are autoinducer-3 (AI-3), which activates virulence genes in enterohemorrhagic *Escherichia coli*, as well as amino acids and short peptides utilized by Gram-positive bacteria. Significantly, QS is essential for planktonic bacteria to transition and adopt the biofilm phenotype [160]. Researchers suggest that various plant extracts and phytochemicals hold considerable promise for addressing bacterial pathogenesis and influencing gut microbiota. In related research, scientists explored anti-QS compounds derived from the secondary metabolites of halophilic marine *Streptomyces*. Their investigation focused on the impact of these compounds on *Proteus mirabilis* biofilm development, revealing encouraging inhibition that suggests they may act as antagonists of bacterial QS [161].

#### 5.1.9 Nano-scale coatings

Nano-scale material in biofilm-associated infection prevention strategies on medical devices has been investigated extensively [13,119,128,137,[162], [163], [164], [165], [166], [167], [168], [169]]. Nanoparticles possess enhanced chemical reactivity and bioactivity when compared to their bulk forms, a characteristic attributed to their high surface area to volume ratio [163]. Their small size allows them to penetrate microbial cell walls, causing the destruction of pathogenic microorganisms attached to the surface. A variety of nanoparticles have been developed and studied to date, encompassing metal and metal oxide nanoparticles, polymeric nanoparticles, and carbon-based nanomaterials like nanotubes and graphene oxide coatings [163,164,167,170]. A good example of the successful implementation of this approach is the sodium-alginate/PAA/PVA hydrogel loaded with 40 nm Ag-NPs, which blocked catheter encrustation for 60 days in a murine CAUTI model and showed <5 % fibroblast viability loss [171]. Another one is the report on a robust double‐network gelatin–polyacrylamide hydrogel containing silver nanoparticles (GPA‐0.01) was developed for Foley‐type latex urinary catheters to improve durability, antibacterial performance, and anti‐biofouling properties [172]. The coating showed strong adhesion, mechanical stability, hydrophilicity, and lubrication, with effective inhibition of *E. coli* on both internal and external surfaces. Antibiofouling was attributed to the combined effects of gelatin and PAAm, while long‐lasting antibacterial activity resulted from gradual silver release. The material also demonstrated good cytocompatibility and blood compatibility, indicating promise for reducing CAUTIs in long‐term biomedical use.

##### 5.1.9.1 Silver NPs

Among coating materials for urinary catheters, silver has received the most research attention because of its substantial exhibition of antimicrobial activity, specifically towards *Staphylococcus aureus* and *Escherichia coli* [167]. Silver nanoparticles (Ag NPs) demonstrate antimicrobial properties by interfering with the division and respiratory systems of microorganisms [167]. According to several authors [119,167] - in contrast to the silver ions, silver nanoparticles exhibit lower toxicity to the host while maintaining similar mechanism of antimicrobial activity. Roe et al. [166] engineered AgNPs (AnNO_3_) coating with sustained release of silver over a period of 10 days. Coated catheter significantly prevent the biofilm formation and showed substantial in vitro antimicrobial activity against numerous pathogens and non-toxic *in vivo* in mice [166].

##### 5.1.9.2 Zinc oxide NPs

Zinc oxide nanoparticles are considered a broad-spectrum antimicrobial material [137,167]. Their ability to kill bacteria stems from several mechanisms: they generate reactive oxygen species, can be internalized by cells, promote the increased uptake of toxic soluble Zn2+ ions, and disrupt cell membranes through electrostatic interactions with the particles [137,167]. Research by Shakerimoghaddam et al. [173] explored the impact of zinc oxide nanoparticles on biofilm formation and the expression of the *flu* gene in uropathogenic *E. coli* (UPEC) strains. Their findings indicated that these nanoparticles inhibited biofilm formation in UPEC isolates. This inhibitory effect was dependent on concentration, with more powerful results observed at minimum inhibitory concentration (MIC) levels compared to sub-MIC concentrations [173]. Even at sub-MIC concentrations, zinc oxide nanoparticles significantly reduced the expression of the *flu* gene in UPEC isolates that were strong biofilm producers. However, these lower concentrations were not sufficient to inhibit biofilm formation directly [173]. Similarly, Sedigheh et al. [174] evaluated the antifungal effects of zinc oxide nanoparticles on *Candida albicans* strains isolated from urinary tract infections. Their investigations revealed that zinc oxide nanoparticles possess a significant capacity to limit both the growth and adhesion of fluconazole-resistant *C. albicans* strains from UTIs, suggesting their potential utility as tools for UTI prevention [174].

##### 5.1.9.3 Polymeric NPs

Polymeric nanoparticles are often employed as nanocarriers for the efficient delivery of antimicrobial agents. This widespread use is attributed to their exceptional stability, high encapsulation efficiency, and the ease with which their surface properties can be modified [167]. In this vein, Ghalei [175] and collaborators demonstrated that silk-based nanoparticles could serve as a biocompatible and cost-effective means to deliver nitric oxide by loading silk fibroin nanoparticles with S-Nitroso-N-acetylpenicillamine. This system exhibited potent antibacterial properties against both MRSA and *E. coli*, crucially without displaying any cytotoxic effects toward fibroblast cells [175]. Furthermore, Srisang et al. [176] developed two types of chlorhexidine-loaded nanoparticles: poly(ethylene glycol)-block-poly(ϵ-caprolactone) micelles and poly(ϵ-caprolactone) nanospheres. These biodegradable polymeric nanoparticles, encapsulating the drug, provided effective antimicrobial activity against *E. coli* and *S. aureus* while simultaneously demonstrating excellent biocompatibility and no toxicity to normal cells [176].

##### 5.1.9.4 Carbon-based nanomaterials

Materials like single-walled carbon nanotubes (SWNT) and graphene oxide (GO), which are types of carbon-based nanomaterials, demonstrate considerable antimicrobial capabilities [137]. Their unique optical and tunable surface properties, along with low systemic toxicities and cost-effectiveness, have led to their widespread application [167]. For instance, Vagos et al. [177] incorporated carbon nanotubes (CNTs) into poly(dimethylsiloxane) (PDMS) to obtain composite material with antiadhesion properties. CNTs incorporation into PDMS resulted in less bacterial adhesion (namely *E. coli*) than on the PDMS alone. Similarly, Anju et al. [178] found rose bengal/multi-layer carbon nanotube conjugate to be effective against *E. coli* biofilm formation. On the other hand, Liu et al. [179] investigated antibacterial activity and mechanism of graphene oxide (GO) coating against *S. aureus* and *E. coli.* They found that the dominant mechanism in antibacterial activity was oxidative stress which resulted in severe damage to *E. coli* and a bit less to *S. aureus.* Despite these points, the authors propose that these coatings, being easily recyclable and presenting no inhalation risk, could represent a feasible option for use on antibacterial medical instruments. While recent studies have shown promising short-term antibacterial activity, the long-term effectiveness and safety of these clinical applications remain uncertain [137].

### 5.2 Relative technology readiness (TRL) of different approaches

**Fig. 7:**
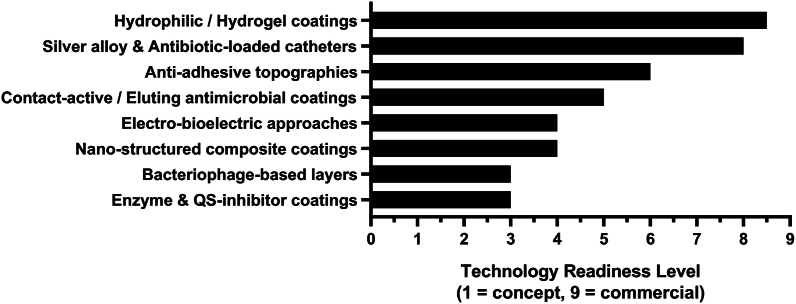
Relative technology readiness (TRL) of different approaches.

Source: own.

Considering the level of commercialization and current use, various proposed solutions are at different TRLs (Fig. 7). For example, hydrophilic/hydrogel coatings are already on the market (TRL 9) and used in many clinical settings, whereas catheters loaded with silver alloys and antibiotics are also routinely used in the short term, but their benefit diminishes after 7 days, necessitating their further improvement (TRL 8). Physical topographies and basic anti-adhesion chemistries currently sit at TRL 6, while most nanoengineering, bioelectrical, and phage-based strategies still remain more or less at the applicative research level (TRLs 3–5), with scale-up and regulatory toxicology being the major hurdles [108,120,135].

### 5.3 Key translational challenges

Several characteristics of catheters that will have to be further improved are still hindering the general uptake of these novel technologies. In the next level in push towards higher TRLs, the following key design specifications will have to be considered.

1. Durability vs. agent release – balancing ≥30-day mechanical robustness with steady, non-toxic antimicrobial flux [120,135].
2. Encrustation control – any coating that ignores *P. mirabilis* urease will fail after ≈ 7–10 days [27,120].
3. Composite biofilms & resistance – mixed bacterial/fungal films require multi-modal approaches (e.g. Ag + Zn + NO) [135].
4. Regulatory path & cost – combination-product status lengthens approval; GMP-compatible, solvent-free self-assembly (e.g. Cu-phenolic) can ease this [136].

## 6 Conclusion

The Global Antimicrobial Resistance and Use Surveillance System report [180] highlights alarmingly high global resistance levels to many commonly used antibiotics for treating infections. Consequently, it's no surprise that the World Health Organization has designated antimicrobial resistance as one of the top ten global public health threats facing humanity. This troubling trend signals a diminishing supply of effective antibiotics. Urinary infections and their causative pathogens are unfortunately not exempt from this growing resistance. As one of the most frequent hospital-acquired infections, they represent a significant challenge to healthcare systems.

Overcoming CAUTI will require a decisive shift from treating established infections to preventing them at the material–microbe interface. This transition demands interdisciplinary cooperation between clinicians, microbiologists, and materials scientists to develop biofilm‐resistant catheters that integrate safety, efficacy, and real‐world usability.

Since novel antibiotics are difficult to develop, it is of the utmost importance to search for new methods to combat these infections. As stated in the previous chapters, there are promising new techniques that incorporate new as well as combine old technologies that are interdisciplinary and can be applied in different fields, not only urinary catheters but also in situations where biofilm formation plays an important role in pathogenesis. Finally, looking at the emergence rate of new resistant antimicrobial strains it is safe to say that interdisciplinary approaches in combination with new materials, and technologies will be critical for battling our problems in the future.

The future of catheter manufacturing is centered on technological innovation and patient-focused advancements, aiming to enhance safety, precision, and comfort. Key trends include the integration of smart sensors for real-time feedback, the use of biocompatible and advanced materials for reduced infection risks and improved flexibility, and the miniaturization of devices to support minimally invasive procedures. Emerging technologies like 3D printing and nanotechnology are paving the way for custom-designed, antimicrobial catheters, while the incorporation of imaging systems ensures precise placement for targeted therapies [181]. Automation and robotics in manufacturing promise consistent quality and scalability, making cutting-edge catheter solutions more accessible and personalized for diverse medical needs. The major targeted technologies for the future focus on multi-layer architectures that (i) deter fouling also “physically”, (ii) enable micro-dosing of broad-spectrum agents on demand, (iii) simultaneously sense pH/urease for real-time blockage alerts, and (iv) stay intact for ≥30 days. Considering this versatile range of properties requires cross-disciplinary consortia that will be critical to translate these prototypes before 2030.

## CRediT authorship contribution statement

**Kristijan Skok:** Writing – review & editing, Writing – original draft, Visualization, Investigation, Conceptualization. **Uroš Bele:** Writing – review & editing, Methodology, Investigation. **Špela Pintar:** Writing – review & editing, Writing – original draft, Investigation. **Zdenka Peršin:** Writing – review & editing, Methodology, Investigation. **Katja Kuzmič:** Writing – review & editing, Methodology. **Matej Bračič:** Writing – review & editing, Methodology. **Lidija Fras Zemljič:** Writing – review & editing, Methodology, Investigation. **Uroš Maver:** Writing – review & editing, Writing – original draft, Visualization, Supervision, Conceptualization.

## Declaration of generative AI and AI-assisted technologies in the writing process

During the preparation of this work the authors used CHAT GPT in order refine the language. After using this tool/service, the authors reviewed and edited the content as needed and take full responsibility for the content of the publication.

## Declaration of competing interest

The authors declare the following financial interests/personal relationships which may be considered as potential competing interests: Uros Maver reports financial support was provided by Slovenian Research and Innovation Agency. If there are other authors, they declare that they have no known competing financial interests or personal relationships that could have appeared to influence the work reported in this paper.

## Data Availability

Not applicable. This is a narrative review that does not involve the collection or analysis of primary data.
