# Supplementary material for: Urinary catheters: state of the art and future perspectives – a narrative review
Source: Mater Today Bio. 2025 Aug 20;34:102225. doi: 10.1016/j.mtbio.2025.102225 (PMC12414918; doi:10.1016/j.mtbio.2025.102225)
Supplement: Multimedia component 1 [file mmc1.docx]

Supplementary Table S1: Comparative summary of treatment strategies for CAUTI Prevention

| **Category** | **Study / Short Ref** | **Ref. #** | **Catheter / Substrate** | **Mechanism** | **Model** | **Duration / Endpoint** |
| --- | --- | --- | --- | --- | --- | --- |
| Hydrogels / Hydrophilic | PVA–chitosan/silver quantum dot hydrogel catheter coating with sustained antibacterial activity | 120 | Latex Foley; PVA/Chitosan double‑network + Ag quantum dots | Hydrophilic anti‑adhesion + gradual Ag release | Dynamic encrustation loop; artificial urine | ≤7 days; blocked encrustation; ~95% urothelial viability |
| Hydrogels / Hydrophilic | Hydrophilic vs PVC catheters | 126 | Hydrophilic‑coated vs PVC UCs | Low‑friction hydrophilic surface | Clinical comparisons | mid term; fewer UTIs |
| Hydrogels / Hydrophilic | Curd‑peptide probiotic hydrogel (QS inhibition) | 122 | Peptide‑based hydrogel | Hydrophilicity + antimicrobial peptides; QS interference | In vitro (S. aureus, P. aeruginosa) | Short‑term; reduced biofilm |
| Anti‑adhesive topography | Impact of engineered surface microtopography on biofilm formation of Staphylococcus aureus | 102 | Micropatterned silicone | Biomimetic microtopography (physical‑only) | E. coli exposure up to 21 days | >40% surface‑coverage reduction vs smooth |
| Anti‑adhesive topography | Influence of Surface Topography on Bacterial Adhesion | 104 | Patterned silicone (varied height/spacing) | Micro/nano‑topography | In vitro (S. epidermidis, B. subtilis, E. coli) | Biofilm density ↓ up to ~45% |
| Anti‑adhesive topography | Effects of Colloidal Crystals, Antibiotics, and Surface-Bound Antimicrobials on Pseudomonas aeruginosa Surface Density | 105 | Hexagonally packed microfeatures on silicone | Topographic deterrence; synergy with antibiotics | In vitro (P. aeruginosa) | ~99% CFU↓; 99.9% with antibiotics |
| Antimicrobial / contact‑active | Dual-Layer Nanoengineered Urinary Catheters with zinc (Zn) and silver nanoparticles (AgNPs) | 135 | NO‑releasing PU + ZnO‑Ag | Diffusible NO + contact‑active metals | In vitro + porcine CAUTI model | Polymicrobial biofilm↓; no added inflammation |
| Antimicrobial / contact‑active | Minocycline + Rifampicin coating | 125 | Antibiotic‑impregnated catheter | Drug elution (Mino+Rif) | In vitro, multi‑species | Prevents Gram± biofilms, P. aeruginosa & Candida |
| Antimicrobial / contact‑active | Copper-phenolic coating | 136 | Metal–phenolic network on silicone | Low‑leach Cu2+ release | In vitro (S. aureus, E. coli) | Rapid kill within ~2 h; negligible hemolysis |
| Zwitterionic / Antifouling polymers | Biomaterials coated with zwitterionic polymer brush demonstrated significant resistance to bacterial adhesion and biofilm formation in comparison to brush coatings incorporated with antibiotics | 109 | Zwitterionic brushes on polyurethane | Non‑fouling hydration layer | 72 h artificial‑urine flow (E. coli, P. aeruginosa) | Biofilm suppressed; non‑cytotoxic |
| Zwitterionic / Antifouling polymers | Highly anionic polysaccharide coating | 121 | Charged polysaccharide on PDMS | Electrostatic repulsion; protein‑/cell‑resistance | In vitro (S. aureus) | Adhesion & growth reduced |
| Zwitterionic / Antifouling polymers | Hydrophilic antimicrobial carboxymethyl chitosan (CMCS) usage | 124 | Chitosan‑based coating | Charge‑balanced non‑fouling | Static & flow; (E. coli, P. mirabilis) | Biofilm suppressed; non‑cytotoxic |
| Stimuli‑responsive / Smart | Colour‑change pH sensors in hydrogels | 73 | Hydrogel with pH dye | Early detection of alkaline crystalline biofilm | Artificial urine; bedside‑detectable | Sensor response with pH shift, capable of signalling infection by P. mirabilis |
| Stimuli‑responsive / Smart | SAW delay of crystalline nucleation | 116 | Piezo‑enabled SAW on catheter tubing | Low‑freq vibrations disrupt adhesion | Dynamic bladder model (P. mirabilis) | cilia were able to efficiently clean typical encrustation |
| Stimuli‑responsive / Smart | Bioelectric effect + SAW sensor/therapy | 114 | Integrated SAW biofilm sensor with therapy | Sensing + electrical disruption | Prototype system; early‑stage | Early detection + active disruption |

Legend: This table summarizes representative studies across major CAUTI prevention strategy categories.

Abbreviations: UC = urinary catheter, CAUTI = catheter‐associated urinary tract infection, AgNPs = silver nanoparticles, QDs = quantum dots, PU = polyurethane, PVA = polyvinyl alcohol, CS = chitosan, QS = quorum sensing.
